# Adaptive capacities from survival to stress responses of two isogenic lines of rainbow trout fed a plant-based diet

**DOI:** 10.1038/srep35957

**Published:** 2016-11-03

**Authors:** B. Sadoul, A. Foucard, C. Valotaire, L. Labbé, L. Goardon, J. M. LeCalvez, F. Médale, E. Quillet, M. Dupont-Nivet, I. Geurden, P. Prunet, V. Colson

**Affiliations:** 1 INRA, UR1037, Fish Physiology and Genomics, 35042 Rennes, France; 2 INRA, UE937 Pisciculture expérimentale des Monts d’Arrée, 29450 Sizun, France; 3 INRA, UR1419 Nutrition Métabolisme Aquaculture, 64310 St-Pée-sur-Nivelle, France; 4 INRA, UMR1313 Génétique Animale et Biologie Intégrative, 78352 Jouy-en-Josas, France

## Abstract

The composition of feed for farmed salmonids has strongly evolved during the last decades due to the substitution of fishery-derived fish oil and fishmeal by ingredients of plant origin. Little information is available regarding the effects of this transition on adaptive capacities in fish. Two rainbow trout isogenic lines, known for their divergent ability to grow on a plant-based diet (PBD), were fed for seven months from first feeding either a fully PBD or a control marine-resources diet and were compared for their growing and survival capacities over time and their behavioral and stress responses at similar sizes but different ages. Although fish displayed similar appetitive behaviour, the two lines were highly affected by the PBD translated in decreased growth and apathetic behaviour, but also stronger stress responses displayed by stronger cortisol increases and more stress-related behaviour when isolated. The two lines were found to be similarly sensitive to a PBD for the assessed stress-related parameters, but one line displayed a lower survival during the early rearing period. Overall, these results suggest that a PBD supplied to fish from the alevin stage has strong effects on physiological and behavioural parameters, with possible impairment of fish welfare, but also genome-dependent survival.

The rapid growth of aquaculture production over the last decades has led to new challenges aiming to improve the sustainability of the sector. One of the major concerns is linked to the reliance on wild fishery resources to feed farmed fish^1^. Scientific efforts are therefore focusing on seeking solutions to decrease this reliance, mostly through substitution of marine-derived fishmeal and fish oil by terrestrial plant resources^2^. Thus, vegetable oils and plant proteins have become in the last years the major sustainable alternative ingredients in aquafeed. But the replacement still remains partial since fully substituted diets reduce growth in species belonging to a high trophic level, like salmonids^3^,^4^. The main cited reasons are decreased feed intake or lower digestibility or altered metabolic pathways observed when replacing marine proteins or marine oil individually^5^,^6^,^7^. However, promising results have been obtained throughout selective breeding, early life experience or new diet compositions in rainbow trout fed the fully substituted diets^8^,^9^. These studies open perspectives to the use, in a near future, of all plant-based diets (PBD) for salmonid feeding practices.

The composition of terrestrial plant ingredients differs from fish oil and fish meal in terms of fatty acid profiles, proteins, amino acids and micro-nutrients contents. In order to adjust concentrations of these essential nutrients to meet fish nutritional requirements^10^, PBD are made of blend of plant sources and supplemented in some amino acids and micro nutrients. However, since none of the terrestrial vegetable oils contains n-3 Highly Unsaturated Fatty Acids (HUFA), the replacement of fish oils by vegetable oils induces unavoidable changes in n-3 HUFA contents, considered as key-components of cell membranes and brain development. Thus, several studies have highlighted the importance of dietary lipids and essential fatty acids in the activation of the HPI axis and release of cortisol^11^,^12^,^13^ or in the stress-related swimming behavior^14^,^15^. A recent study however, did not show major physiological nor behavioural modifications in sea bass fed a PBD introduced at early stage^16^. To our knowledge the effects on fish behaviour, stress responses and welfare of a total replacement of fishmeal and fish oil by plant ingredients have never been investigated in salmonids, despite the major economic concern of these farmed species.

In this study, we compared the effects of a marine diet (MA) containing fish oil and fishmeal and a fully substituted plant-based diet (PB) free from marine ingredients on rearing capacities, behavioural parameters and stress responses using a standardized protocol previously used in rainbow trout^17^. Furthermore, the effect of the genotype/diet interaction on these parameters was assessed by running the experiments with two isogenic lines of rainbow trout previously characterized for their different ability to grow during a PBD period^9^ and showing divergent responses to an acute stress^18^.

## Materials and Methods

### Ethical statements

Fecundation, hatching, rearing and experimental procedures were performed at the PEIMA experimental farm (INRA, Sizun, France) having authorization for experimentation (agreement number: C29-447-02). All experimental procedures used in this study were approved by the local Ethic, Animal Care and Use Committee of INRA-PEIMA provided by the French legislation under the official licence N°74. They were performed in respect of the Guide for the Care and Use of Laboratory Animal edited by the National Council for Animal Experimentation of the French Ministry of Higher Education and Research and the Ministry of Food, Agriculture and Forest.

### Isogenic lines

Two heterozygous isogenic lines of rainbow trout (*Oncorhynchus mykiss)* were produced by INRA-GABI (Jouy-en-Josas, France). The homozygous lines used as broodstock were previously established after two generations of gynogenesis and further maintained by within line single pair mating using sex reversed XX males^19^. The eggs from two fully homozygous females (having the same genotype - two females were used to obtain enough eggs), from a single maternal line, were mixed and redistributed in two batches. The eggs were then fertilised using two homozygous sires from different lines (A22 and R23) producing heterozygous lines, mentioned as A and R in this manuscript. Therefore, genetic differences between the two fish lines can be attributed to paternal differences, and all individuals within one line are expected to share the same genotype. Sires and dams were controlled to check for homozygosity and conformity with the original line using 10 microsatellites. Isogenic line R was shown in previous unpublished studies to be particularly performant in terms of growth when fed a plant based diet from first feeding.

### Rearing history

Similarly to those described previously^9^, MA and PB diets were manufactured at INRA’s facility of Donzacq (France) using a twin screw extruder (Clextral, France). Diets compositions are shown in Supplementary Table S1. Briefly, MA diet contained fishmeal and fish oil as protein and lipid sources, respectively. PB diet contained a mix of plant protein sources and a blend of palmseed, rapeseed and linseed oil as lipid source. Careful attention was given to avoid exceeding anti-nutrients in the PB diet that was further supplemented with the essential amino acids, lysine and methionine, and micronutrients in order to fulfil the known nutrient requirements of rainbow trout.

After vitellus resorption, at 52 days post-fertilisation (dpf), fish from each line were transferred to distinct tanks (400 fry/0.3 m^3^), being fed ad libitum either with the MA diet or with the PB diet (n = 3 tanks per condition). Thus, in total, 12 tanks were used, according to the 4 conditions: line A fed the MA diet (AMA), line A fed the PB diet (APB), line R fed the MA diet (RMA) and line R fed the PB diet (RPB). All tanks were kept under identical rearing conditions using a flow-through system supplied with natural spring water (11.4 °C) until 118 days post fertilisation, and Drennec lake water beyond (11.5–18 °C). From 152 dpf onwards, one more tank was used for each condition, in order to cope with the increasing density (n = 4 tanks per condition).

### Experimental design

#### Rearing performances

Throughout the rearing period, eight weight measures were performed on each tank. Weight was assessed by group, by netting, counting and weighting around 20% of the fish from a tank. Additionally, every tank was daily observed, and dead fish were recorded and removed. The sum of dead fish over the period between two weight measures was analysed.

#### Experimental set-up

Growth was significantly different between conditions (see results section). In order to partially compensate for weight differences, conditions were tested in a sequential order starting with RMA (248 dpf), followed by AMA (255 dpf), RPB (290 dpf) and APB (297 dpf) (see Supplementary Table S2). This sequential experimental protocol enabled to test all groups at similar weights (ranging from 58.7 g to 83.2 g). Supplementary Table S2 shows fish weight at the start of the experiment for each condition, with corresponding mean water temperature over the 17-day experimental period.

For the experimental phase, eight experimental tanks were used per condition, using 40 fish per experimental tank. Each experimental tank (0.15 m3, rectangular, uncovered) was equipped with a video camera placed above. The tanks were supplied with the Drennec lake water. Luminosity was controlled and tanks were automatically illuminated from 8:00 to 17:00. Fish from each tank were fed twice daily (at 11:00 and 16:00) using automatic feeders (Arvo-Tec^®^, TD 2000, 2 rpm).

One week before the experimental phase, fish were first transferred to 0.3 m^3^ covered acclimation tanks in order to acclimate the fish to the experimental conditions.

#### Fish group behaviour

After one week in the experimental tank (day 1), fish were filmed at days 1, 3, 8 and 10. In the rest of this paper, this period will be called “the pre-challenge period” (day 1–10). For each of these days, 2 videos of 5 minutes each at 8:30 and 16:30 and one video of 5 min before and 5 min after the first food distribution at 11:00 were recorded. In videos recorded at 8:30 and 16:30, the following behavioural parameters were counted in each tank using the focal sampling method:

- Stereotypies, defined as repetitive swim against the edge of the tank. Each act was manually counted as an occurrence.
- Aggressive acts defined as bite attempts and pursues through the tank. This parameter was counted when a fish moved away from its initial position with a propulsive tail stroke, immediately followed by another fish.

In videos recorded at 11:00, the fish shoal activity was assessed. For that purpose, one image every 0.5 second over the 10-minutes videos was analysed using the tool described by Sadoul and coworkers^27^. The tool was modified in order to also automatically measure the percentage of fish near the feeder before and after the meal (Supplementary Method).

#### Individual emotional reactivity and cortisol response

After the pre-challenge period (day 11), the behavioural and the cortisol responses of fish isolated in a new environment were assessed using an individual emotional reactivity test (ER test). This test consisted in catching simultaneously 4 fish (2 individuals per tank, n = 16 fish per condition) and immediately transferring them in 4 novel individual tanks (1 × 1 × 0.80 m). Then the experimenter left the area and immediate behavioural responses of each isolated fish were video-recorded for 20 minutes (sampling rate of 5 pictures per second) and analysed using Ethovision software (Noldus, Wageningen, The Netherlands). In the software, the tank containing the experimental fish was divided into two areas, a central zone (0.6 × 0.6 m) and a 20 cm border zone. The following behavioural parameters were calculated in Ethovision for each individual: mean swimming speed (cm·s^−1^), maximum swimming speed (cm·s^−1^), percentage of time spent in movement (%) (starting when velocity was higher than 2 cm·s^−1^), percentage of time spent in the border zone - defined as thigmotaxis (%), and angular velocity (°·s^−1^).

For cortisol analyses, blood from 8 fish of each condition was collected before the ER test, for baseline cortisol. Blood samples were also collected from 8 tested trout 30 and 90 minutes after the beginning of the ER test. The fish were netted and transferred to buckets containing the circuit water to which 2-phenoxyethanol (0.1% vol) had been added for euthanasia. Blood was sampled from caudal sinuses into heparinized syringes and samples were stored on ice. After sampling, blood cells and plasma were separated by centrifugation (10 minutes at 3000 rpm). Plasma was collected and frozen at −20 °C until analysis. Before the cortisol assay, steroids were extracted twice from 50 μl plasma with 2 ml ethylacetate/cyclohexane. After solvent evaporation, steroids were suspended in 250 μl assay buffer. Cortisol was assayed in duplicate by a ^3^H cortisol radioimmunoassay according to the method described by Auperin *et al*.^21^ and Colson *et al*.^29^.

### Statistical analyses

Results were analysed using the software R3.1.3 (http://cran.r-project.org/). The last weights before the first set of acclimation (234 dpf) were compared between the fish line and the diet using a 2-way ANOVA. Observations obtained with focal sampling during the pre-challenge period were repeated during 4 days on the same tanks. For these data, we used therefore a generalised linear mixed model with the fixed effects line, diet and day and the 2-way and 3-way interactions. Tank effects considered as random were also included. Morning and afternoon data obtained for stereotypies and aggressive behaviour were analysed separately. The distribution of the aggressive behaviour was specified to follow a Poisson distribution. The measures of stereotypies were showing a distribution too far from the Poisson distribution and were therefore rank-transformed before running the analysis using the same mixed model.

For data concerning the proximity to the feeder, the means before and after feeding were calculated for each tank and each day and analysed separately using the same linear mixed model. These data were transformed using arcsin to be in accordance with the normal distribution of the residuals. Similarly, mean activity before feeding was calculated and analysed using the same model.

Results for the individual emotional reactivity were not repeated over time and could therefore be analysed using simple linear models with line, diet and interaction as fixed effects, followed by an ANOVA. The effect of fish length was tested for all these measures but was never found to be significant, and was therefore removed from the linear models. Only the measure of the time spent in thigmotaxis was rank-transformed to be in accordance with the normality assumption.

For all models, the non-significant interactions were removed and the models were run again. Post-hoc tests were run within each day or sampling time (for cortisol) using a Tukey HSD test.

## Results

### Survival and growth during the rearing period

**Figure 1:**
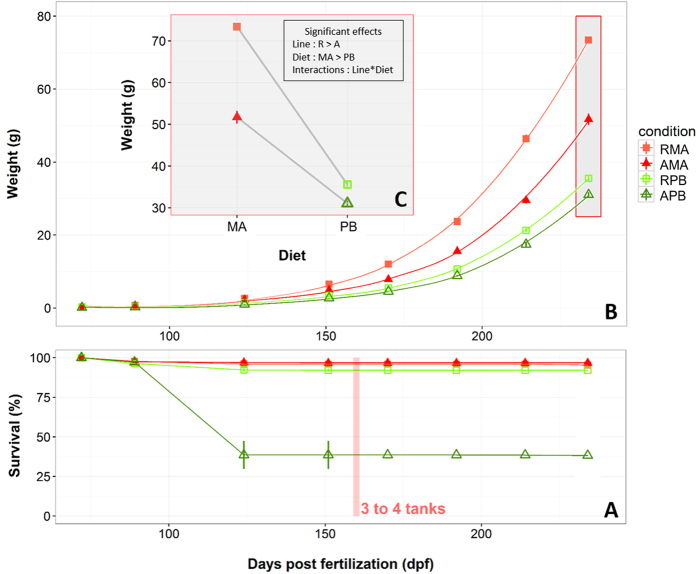
Survival over time (**A**), body weight over time (**B**) and body weight at the first acclimation period (234 days after fertilisation) (**C**) in two isogenic lines (A and R) of rainbow trout fed a marine (MA) or a plant-based diet (PB). The mean and error bars are presented for each condition (n = 3–4 replicated tanks). Lack of vertical bar indicates SE smaller than symbol size. A local polynomial regression fitting was used to produce the growth curves in B. Before 151 dpf, 3 tanks were used per condition. After 151 dpf, fish from the 3 tanks were mixed and randomly distributed in 4 tanks per condition in order to deal with increasing density.

Figure 1A illustrates fish survival over time for the four conditions, and Supplementary table S3 provides the mean data of weight, weight gain, survival, Food Conversion Ratio and Specific Growth Rate for each condition over time. APB-fish showed a strong mortality episode between 89 and 124 dpf in the three rearing tanks, with mean survival at 124 dpf dropping down to 38%, significantly different (*p* < 0.0001) from all other conditions. RMA-, AMA- and RPB-fish showed during the same period a mean survival above 90%. This sudden mortality coincides with an increase in SGR and a drop in FCR for APB (Supplementary 4). After this mortality episode, APB-fish showed a normal survival until end of experiment and FCR and SGR were similar to the other conditions (Supplementary 4). Figure 1B illustrates growth pattern overtime, with RMA-fish showing always the highest weights, followed by AMA, RPB and APB. Statistical analyses performed at 234 dpf showed that fish from line R were overall heavier compared to fish from line A, and that the plant-based diet had a negative effect on fish weight in both lines (Fig. 1C). Additionally, a significant interaction was found, with line A being less impacted by the plant-based diet than line R, with a respective weight decrease of 40 and 51.6% linked to the PB relative to the MA diet.

### Behavioural observations

**Figure 2:**
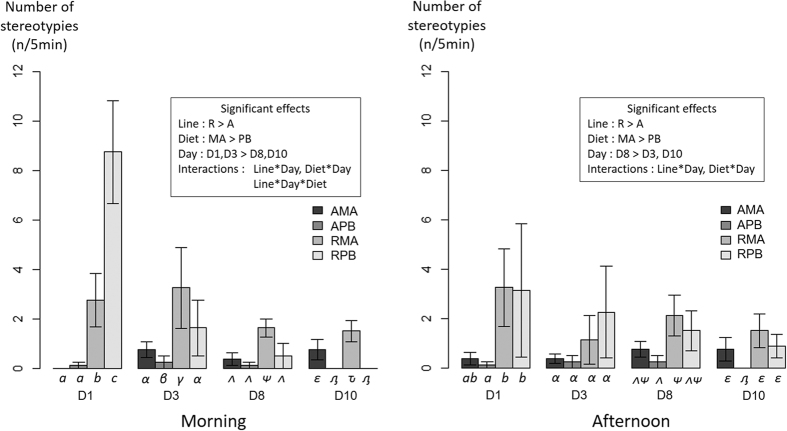
Number of stereotypies counted for 5 minutes in the morning or the afternoon during pre-challenge period in two isogenic lines (A and R) of rainbow trout fed a marine (MA) or a plant-based diet (PB). The mean and error bars are presented for each condition (n = 8 replicated tanks). Significant main effects and interactions (P < 0.05) are indicated. Multiple pairwise comparisons were performed within each day. Conditions with different Latin letters, Greek lowercase letters, Greek uppercase letters and Cyrillic letters under the bar are significantly different within day 1, 3, 8 and 10 respectively.

**Figure 3:**
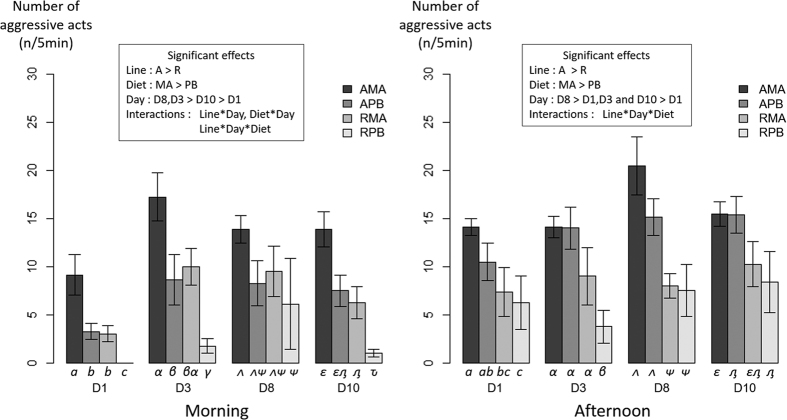
Number of aggressive acts counted for 5 minutes in the morning or the afternoon during pre-challenge period in two groups of isogenic lines (A and R) of rainbow trout fed a marine (MA) or a plant-based diet (PB). The mean and error bars are presented for each condition (n = 8). Significant main effects and interactions (P < 0.05) are indicated. Multiple pairwise comparisons were performed within each day. Conditions with different Latin letters, Greek lowercase letters, Greek uppercase letters and Cyrillic letters under the bar are significantly different within day 1, 3, 8 and 10 respectively.

**Figure 4:**
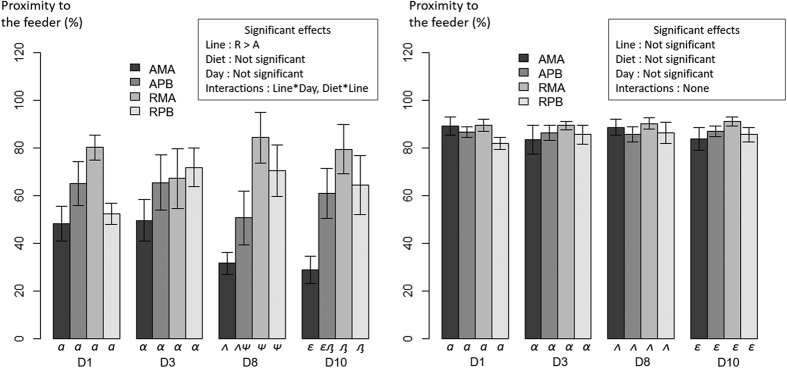
Percentage of fish recorded during pre-challenge period in the area close to the automatic feeder 5 minutes before and 5 minutes after feed distribution (at 300 s) in two isogenic lines (A and R) of rainbow trout fed a marine (MA) or a plant-based diet (PB). The mean and error bars are presented for each condition. Significant main effects and interactions (P < 0.05) are indicated. Multiple pairwise comparisons were performed within each day. Conditions with different Latin letters, Greek lowercase letters, Greek uppercase letters and Cyrillic letters under the bar are significantly different within day 1, 3, 8 and 10 respectively.

Behavioural observations were performed over 10 days following a week of acclimation in the experimental tanks. The effect of isogenic line, day of pre-challenge, diet and their interactions on these behaviours are described in Figs 2, 3, 4 and 6. There was a significant effect of the isogenic line on the number of stereotypies and aggressive behaviour (Figs 2 and 3). Globally, line A was displaying less stereotypies (Fig. 2) compared to line R but seems to be more aggressive (Fig. 3). A strong day effect was found, suggesting that acclimation continued during the pre-challenge period. This was very clear with the number of stereotypies showing a strong decrease over time in the morning. The dietary treatment had also a significant effect on the two behavioural observations, with fish fed the PB diet showing an overall lower number of stereotypies and reduced aggressive behaviour (Figs 2 and 3). The interactions Line*Day and Diet*Day were significant for the two behavioural parameters, indicating that the acclimation capacity to the experimental tank is line and diet dependent (acclimation speed different). On the contrary, the interaction Line*Diet was never significant suggesting that the two lines show a similar behavioural response to the diet. However, a significant 3-way interaction was found for the stereotypies and aggressive behaviour in the morning (p < 0.05; Figs 2 and 3). These results indicate that depending on the line, the diet has an effect on the behavioural consistency throughout the days. This is very clear with the number of stereotypies, where line R with PB diet was showing a stronger decrease during time compared to other conditions.

**Figure 5:**
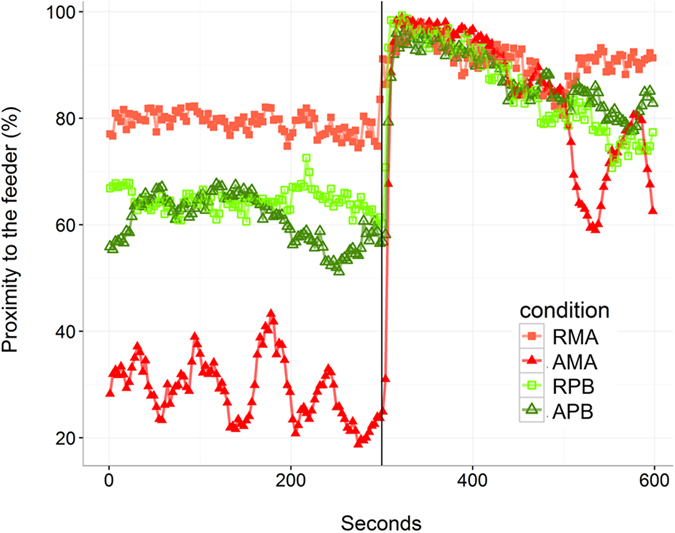
Percentage of fish recorded on day 10 in the area close to the automatic feeder 5 minutes before and 5 minutes after feed distribution (at 300 s) in two isogenic lines (A and R) of rainbow trout fed a marine (MA) or a plant-based diet (PB). The mean of all tanks are presented without the error-bars for clarity. Statistical analyses are presented in Fig. 6 for all 4 days of observation.

The statistical analysis performed for the proximity to the feeder showed a significant effect of the line prior to feeding, with line A showing lower values than line R (Fig. 4). Moreover, a significant interaction Line*Diet was found, with both lines consuming the PB diet showing an intermediate response prior to feeding, in between the two MA dietary groups responses prior to feeding. These differences became non-significant after the feeding, indicating that the four conditions displayed the same feeding activity after food distribution. Immediately after the meal, all conditions were showing a similar pattern with a very quick movement to the food area, illustrated in Figure 5. No significant three-way interaction was found suggesting that the diet does not have an effect on the line acclimation pattern and vice versa for this parameter.

**Figure 6:**
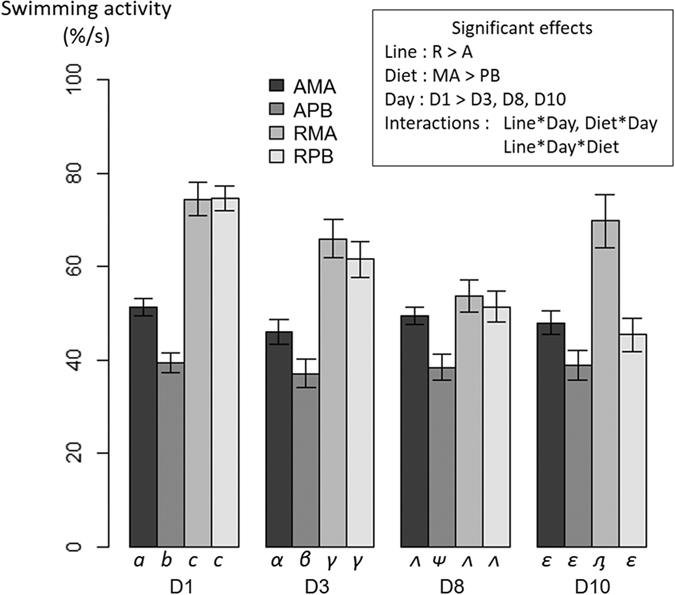
Mean swimming activity of the shoal in tank for two isogenic lines (A and R) of rainbow trout fed a marine (MA) or a plant-based diet (PB) during 5 minutes prior to feeding. The mean and error bars are presented for each condition. Significant main effects and interactions (P < 0.05) are indicated. Multiple pairwise comparisons were performed within each day. Conditions with different Latin letters, Greek lowercase letters, Greek uppercase letters and Cyrillic letters under the bar are significantly different within day 1, 3, 8 and 10 respectively.

The swimming activity prior to feeding for each condition and each day is shown in Fig. 6, illustrating that the PB diet has globally a negative effect on the activity and that line A is less active than line R, as confirmed by statistical analyses. Again, significant interactions Line*Day and Diet*Day were found, suggesting that this behavioural parameter was also not totally fixed over time during the pre-challenge period. This was particularly true with conditions RPB and RMA showing important modifications during this period, whereas line A was showing an overall more consistent behaviour. Moreover, the 3-way interaction was significant and was illustrated by the difference in response during the period between RPB and RMA, with RPB showing a clear decrease in activity over time whereas no clear pattern was seen in RMA.

### Individual emotional reactivity and cortisol response after the pre-challenge period

**Figure 7:**
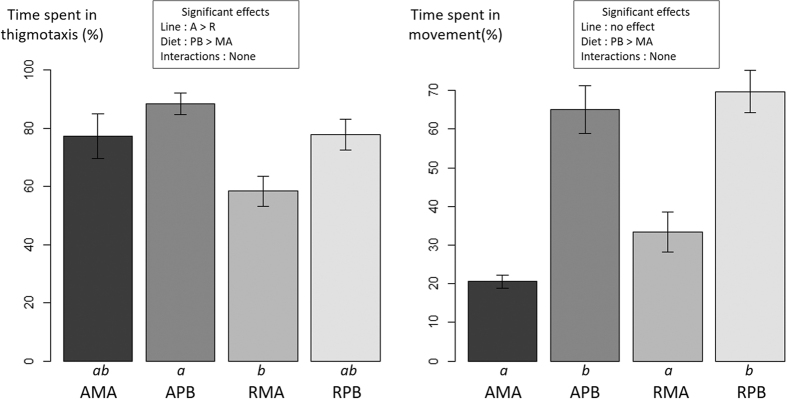
Behavioural responses during emotional reactivity to isolation in a novel environment in two isogenic lines (A and R) of rainbow trout fed a marine (MA) or a plant-based diet (PB). Significant main effects and interactions (P < 0.05) are indicated. Conditions with different letters under the bar are significantly different.

Time spent in thigmotaxis and time spent in movement were both significantly higher in groups fed the PB diet (Fig. 7). Moreover, line R spent significantly less time in thigmotaxis than line A. No significant interaction Diet*Line was found for both behaviours.

**Figure 8:**
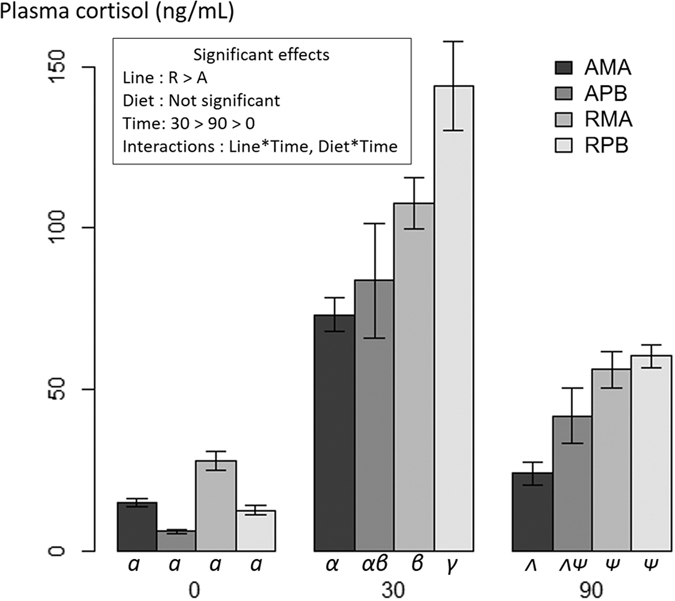
Plasma cortisol before (0 min) and after an isolation stress for 30 or 90 minutes in two isogenic lines (A and R) of rainbow trout fed a marine (MA) or a plant-based diet (PB). Significant main effects and interactions (P < 0.05) are indicated. Multiple pairwise comparisons were performed within each time. Conditions with different Latin letters, Greek lowercase letters and Greek uppercase letters under the bar are significantly different within time 0, 30 and 60 respectively.

A significant time effect was observed on plasma cortisol levels in response to the isolation stress, indicating that the HPI axis was triggered (Fig. 8). A statistically significant line effect was observed with overall higher cortisol levels in fish from line R. A Line*Time interaction was observed, translating a difference in the dynamic of cortisol response between the two lines. Similarly, the significant Diet*Time interaction suggests that the diet had an effect on the cortisol response to the stressor, with the lines fed the PBD showing a stronger increase in their cortisol values. Interestingly, no significant Line:Diet:Time interaction was found, indicating that the plant-based diet had the same effect on the cortisol dynamic in response to the stressor in the two lines.

## Discussion

The need to substitute fishery-derived fishmeal and fish oil by more sustainable ingredients in the feed of aquaculture farmed fish has resulted in a shift to plant-based diets for carnivorous fish like rainbow trout. A fully substituted diet is conceivable if survival, growth, health and welfare of the fish are maintained. This study describes the effects of a fully plant-based diet on all these parameters. The effects have been evaluated in two isogenic lines of rainbow trout.

### Two highly divergent isogenic lines

The two isogenic lines A and R were chosen based on their phenotypic divergences, as previously described^9^,^18^. The present study shows again important differences between the two lines independent of the type of diet, with line A showing slower growth^9^ but also higher aggressive behaviour, less stereotypic behaviour and lower activity when reared in groups. The behavioural results are consistent with the results from Sadoul *et al.*^18^ for aggressiveness and activity (stereotypies were not evaluated), despite the difference in weight of the fish between the two studies (60–80 g in the present vs 2–5 g in the previous study). Indeed, visual observations by means of videos show in both studies a clear dominance effect in line A with all fish condensed in one area of the tank except for one or two dominant fish freely swimming in the tank and performing aggressive acts against the others. In the present study, the high dominance expressed in line A explains the small number of fish observed close to the feeder before feeding. The dominant fish clearly prevented the others to approach the feeding area but when feeding occurred, the alpha fish could not contain the group anymore. This phenomenon was more obvious in the AMA than in the APB group, translated by significant reduction in fish locomotor activity in fish fed the plant-based diet (cf. next discussion section). The slight decrease in feeder area occupancy occurring 200 seconds after feeding is most likely due to recurring aggressive acts attempted by the dominant, chasing again the group at the opposite side. The causes for the emergence of a dominant fish, as observed in the replicate tanks of the isogenic line A, still need to be explained. But it is particularly interesting to highlight in our study that uncontrolled effects are leading to the formation of a dominant fish within a group of individuals supposed to be genetically homogenous. This phenomenon might be due to slight differences in fish life histories rather than genetic differences between them, leading for instance to growth gaps associated with the formation of a hierarchic structure in the group.

These results, combined with those from Sadoul *et al.*^18^, hence show an intra-line consistency for stereotypies and aggression throughout different life stages. Moreover, the between-line differences were not altered by feeding the PBD (no Line:Diet interactions), indicating a consistency in the behavioural profile irrespective of the dietary condition. Activity and aggressiveness can therefore be considered as personality traits as previously described^23^,^24^,^25^. Concerning the plasma cortisol, our study reveals a lower amplitude in response to a stressor for line A, independent of the type of diets. This is consistent with the concept of coping style, which considers that aggressiveness is correlated with a low corticosteroid response to an acute stress^26^. However, these results are in contradiction with our previous work where line A was showing a stronger HPI reactivity when exposed to a confinement stress^18^. The HPI reactivity is nevertheless known to be highly repeatable and consistent between generations, and ages^27^,^28^,^29^,^30^. Yet, it is important to note that all these studies looked at the repeatability of the stress response of fish using the same stressor, whereas different stressors were used between our two studies. Indeed, while in the present study cortisol response is assessed after an emotional reactivity test, a confinement stress in group was used by Sadoul *et al*.^18^. We therefore hypothesize that the HPI reactivity depends on the type of stressor; the cortisol response to isolation in a novel environment is not necessarily correlated to the cortisol response to a confinement stress. Further work is needed to clarify the importance of the type of stressor on the coping style in rainbow trout.

### The plant-based diet modifies growth behavioural and physiological parameters

The change in diet composition had a significant effect on growth but also on most of the behavioural and physiological parameters measured, independently of the fish line. The proximity to the feeder before and after feeding was the only parameter which was not significantly affected by changes in diet composition. After the food distribution, all conditions were showing the same values for the proximity to the feeder suggesting that appetitive behaviour was not impaired by the plant-based diet. These results do not imply identical intakes but advocate that fish were not chronically stressed when fed the plant-based diet^31^,^32^.

A nature-based definition of fish welfare puts forward the idea that a good welfare is illustrated by the expression of natural behaviour by the animal^33^,^34^,^35^,^36^. In our study, it was assumed that fish fed the marine diet would express more natural basic behaviour since the diet is nutritionally closer to what they can find in nature and since it results in an overall better growth. The marine diet condition can thus be considered as the control condition. Under this hypothesis, the strong diet effect observed would suggest that fish are negatively affected by the plant-based diet. All the behavioural and physiological differences compared to the marine diet might then be considered as abnormal, revealing an altered welfare under plant-feeding conditions. This conclusion and its associated assumptions need however to be discussed based on the analysed parameters. Indeed, in both lines, fish fed the plant-based diet showed a decrease in the number of stereotypies and aggressive behaviour. Although more and more criticized, stereotypies are often considered as unnatural and indicative of frustration in vertebrates with an increase in the number of stereotypies translating a decrease in welfare^34^,^37^,^38^. Similarly, a high aggressive behaviour often leads to altered fish welfare with negative consequences like fin damages, impaired immune functions or decrease in food intake and increased energy expenditure^39^,^40^. As such, the observed reduction of aggressive behaviour and stereotypies by the plant-based diet would thus reflect improved welfare compared to the classical marine diet. We however believe that this effect instead indicates a global decrease in energy expenditure in plant-fed fish showing a more apathetic behaviour leading to a general trend for less movements and activity, thus suggesting a negative impact on fish welfare^41^. These data illustrate the importance of having a multi-parameter analysis involving behavioural, physiological and zootechnical parameters when assessing fish welfare^42^. Our study is the first to analyse behavioural parameters within a group of fish fed a plant-based diet. Social behaviour is an important indicator of fish welfare since observations are performed under near aquaculture conditions and in a non-stressed state. A previous study on sea bass fed a fully substituted diet from 40 days after the first feeding, failed to show any effect of the diet on the behaviour of isolated fish^16^. The isolation of fish is a particular context, which can potentially erase differences between individuals due to extreme conditions. Group behavioural data on aggressiveness, stereotypies or activity in unstressed fish might have led to different conclusions. The need for multi-parameter studies analysing not only a stressed individual but also the group in non-stressed conditions is therefore essential to correctly assess welfare^35^,^41^,^43^.

Interestingly, the present study showed also a significant effect of the diet on the stress-related responses when measured individually. Individual trout isolated in a new environment were spending more time in movement, more time close to the wall of the tank (thigmotaxis), and also showed a stronger cortisol increase to isolation stress when fed the plant-based diet compared to the marine diet. This relationship observed between cortisol release after the isolation test and behavioural emotional responses is consistent with the literature linking fearfulness and high post-stress cortisol levels^44^. The open-field test is commonly used in mammals^45^,^46^ and also in fish^17^,^47^ to reveal emotional states and behavioural coping strategies displayed in potentially threatening situations^48^. Increased thigmotaxis and excessive movements observed in fish fed the plant-based diet in response to isolation can respectively express hiding and escaping strategies, which are both anti-predator behaviour. Emotional, physiological and behavioural responses are known to be modified by chronic stress^49^,^50^. Here, the plant-based diet induced the over-expression of fear-related behaviour and higher post-stress cortisol response compared to that of individuals from the marine-diet control group, which hints at the plant-based diet as a possible disturbance in trout. Several studies in fish indicate that highly unsaturated long-chain omega 3 fatty acids such as docosahexaenoic (DHA), present in fish oil but not in plant oils, are crucial in stress responses and brain development^14^,^51^. Pike perch larvae tested individually and fed diets low in DHA exhibit more time swimming along the edge of the arena and have overall higher locomotor activities compared to larvae fed a diet with high DHA content^52^. In mammals, it is well known that dietary DHA deficiencies lead to elevated vulnerability to stress and also decrease cognitive functions (reviewed by Dyall *et al.*^53^). Our results thus show that the trout lines fed the plant-diet, apparently apathetic in group, actually express a stronger emotional reactivity when subjected to an acute isolation stress, which is in total accordance with the study performed on pike perch^52^. Moreover, the plasma cortisol dynamic in response to the isolation stressor show a more important cortisol increase (significant Diet*Time interaction) in trout fed the PBD, suggesting that the PBD-fed fish present a stronger HPI sensitivity in line with other previous studies looking at the effect of changes in dietary fatty acid supply due to fish oil replacement in seabass and seabream^54^. Plant-based diets are also known to be slightly deficient in some specific amino acids such as tryptophan, the major precursor of serotonin. This essential amino acid plays a key role for stress coping and neuroendocrine control^55^,^56^. Similar studies on fish stress-related behaviour, examining the effects of specifically targeted components in plant-based diets, would be of real interest to link the nutritional investigations with those on fish welfare.

Overall, the fully plant-based diet led to more apathetic group behaviour, and increased emotional reactivity in both isogenic fish lines, effects possibly due to changes in the polyunsaturated fatty acid profile and/or essential amino acid balance, or due to the presence of some anti-nutritional factors (from e.g. lupinseed or soybean). These results suggest therefore a general negative impact of the plant-based diet on rainbow trout welfare. However, in our protocol, fish from the two different diets were analysed at a similar size but with one month gap. One cannot formerly exclude the possibility that differences in life-history between the two diets could not be responsible for the differences in stress-related results, such as cortisol or behavioural responses as recently shown in seabass^57^. Additional results are necessary to provide a complete demonstration of the effects of a plant-based diet on these parameters, by using fish of the same age, with, nevertheless, the counterpart of comparing fish of different size also known to impact stress responses^58^.

### Are isogenic lines differently impacted by the plant-based diet?

One of the aims of this study was to investigate whether both genetic lines show the same rearing capacities and stress-related responses to the PBD or, in other terms, whether one line appears more resistant to changes in ingredient source.

Our results showed a significant interaction Diet*Line for survival, with line A fed a PBD showing a strong mortality in the three rearing tanks. This result suggests that line A is more vulnerable when fed a PBD, but also that line R shows a stronger adaptive capacity. Overall these results are arguing in favour of a better robustness of line R, as previously hypothesized based on stress response reactivity and variability to a confinement challenge^18^. The sudden mortality episode observed between 100 and 124 dpf for APB-fish created an artificial selection. The drivers of this selection still need to be described, but the sudden jump in SGR and FCR suggest that the most efficient fish were selected. However, the use of isogenic lines permits to avoid any genetic shift due to mortality, with APB population having the same genome before and after the mortality event. Further work needs to be performed in order to prove whether the selection is dependent upon epigenetic modifications.

Our results showed a significant Diet:Line interaction for the weight at the end of the 234 days rearing period, reporting divergences in the ability to grow under a plant-based diet between the two lines. A previous study on 7 different isogenic lines of rainbow trout also reported similar interactions for feed efficiency, food intake, growth and survival^59^. In our study, the interaction suggests that line A is less impacted by the plant-based diet compared to line R, with a decrease in weight lower for line A than line R. These line-effects are consistent with a previous study investigating the effect of a 25-day challenge with a plant-based diet according to the early-life dietary experience in both lines^9^. These results suggest that line A shows, after the selection process, a lower sensitivity to the dietary changes in terms of growth, arguing in favour of a strong energy allocation to maintain a correct growth in line A fed a plant-based diet. As described in energetic trade-off theories^60^, such energy expenses are concomitant with decreased energy available for other functions, including coping abilities, leading to a trade-off between growth and survival in a changing environment.

Interestingly, as indicated by the overall absence of a significant Diet*Line interaction on the measured behavioural and physiological parameters, no clear difference could be detected between the two lines which displayed the same behavioural and physiological plasticity when fed the plant-based diet. Therefore, the differences of sensitivity observed for growth and survival cannot be explained by differences in behavioural and physiological parameters in this study. Whether this is also observed in fish having different sizes but similar life histories still need to be addressed.

## Conclusions

This study confirms results from a previous study^18^ showing different behavioural phenotypes between two rainbow trout isogenic lines. More importantly, this study also highlights strong effects of the plant-based diet on physiological and behavioural welfare parameters in rainbow trout. Growth and survival data discriminated the two lines in terms of their life history strategies when fed a plant-based diet. However, the two lines were similarly impacted by this diet in terms of stress sensitivity and welfare parameters, failing to discriminate a more resistant genotype able to face nutritional deficiencies for these biological traits. Our results were obtained in two isogenic trout lines: it seems therefore important now to confirm these results with commercial trout having the same life history.

## Additional Information

**How to cite this article**: Sadoul, B. *et al.* Adaptive capacities from survival to stress responses of two isogenic lines of rainbow trout fed a plant-based diet. *Sci. Rep.* **6**, 35957; doi: 10.1038/srep35957 (2016).

**Publisher’s note**: Springer Nature remains neutral with regard to jurisdictional claims in published maps and institutional affiliations.

## Supplementary Material

Supplementary Information
