# Supplementary material for: Adaptive capacities from survival to stress responses of two isogenic lines of rainbow trout fed a plant-based diet
Source: Sci Rep. 2016 Nov 3;6:35957. doi: 10.1038/srep35957 (PMC5093906; doi:10.1038/srep35957)

Supplementary information for the manuscript:

Adaptive capacities from survival to stress responses of two isogenic lines of rainbow trout fed a plant-based diet

Sadoul B.[1,2*], Foucard A.[1], Valotaire C[1], Labbé L.[3], Goardon L.[3], LeCalvez J.M.[3], Médale F.[4], Quillet E.[5], Dupont-Nivet M.[5], Geurden I.[4], Prunet P.[1], Colson V.[1]

[1] INRA, LPGP Fish Physiology and Genomics UR1037, Rennes, France

[2] Current address: Department of Biological Sciences, University of Calgary, Calgary, Alberta T2N 1N4, Canada.

[3] INRA, UE937 Pisciculture expérimentale des Monts d'Arrée, F- 29450 Sizun, France

[4] INRA, UR1067 Nutrition Métabolisme Aquaculture, F-64310 St-Pée-sur-Nivelle, France

[5] INRA, UMR 1313 Génétique Animale et Biologie Intégrative, 78352 Jouy-en-Josas, France

[*]Corresponding author. Email address: bastien.sadoul@ucalgary.ca Phone number: +14038610829

Supplementary method

Automatic shoal activity and proximity to the feeder measures

The fish shoal activity was also assessed. For that purpose, one image every 0.5 second was extracted from the 10-minute videos and was analysed using a modified version of the tool described by Sadoul and co-workers (2014, *Aquaculture*, **430**-179-187). This tool developed on the free software ImageJ is able to automatically analyse images from tank in near aquaculture conditions to estimate the activity and the dispersion (not used in this paper) of the fish. Briefly, the tool first normalizes each image by a background image of the empty tank and transforms the results in a binary image in order to show the fish in black and the background in white. Shoal activity is then assessed by calculating the difference of dark areas between two consecutive images. This difference is proportional to the movement of the fish within the time lapse between the two images. The difference is then divided by the total area of the fish when they were not overlapping in order to take into account the between tank variability in fish size. The results are expressed as a percentage of change per second.

The tool was modified in order to also automatically measure on binary images the percentage of fish near the feeder before and after the meal. To assess the proximity to the feeder, the tank was divided in two complementary areas with one half of the tank on the feeder side and one half on the opposite side. The dark area (corresponding to the fish) in each side of the tank was then measured, enabling to calculate the percentage of fish observed near the feeder.

Supplementary S1. Diet composition of the control marine-resource diet (MA) and the plant-based diet (PB).

Ingredients (g 100 g^{-1} diet)	MA	PB
Fish oil	14,4	-
Plant oil blend*	-	17,5
Fishmeal LT	58,8	-
White lupinseed meal	-	7,2
Corn gluten meal	-	18,0
Soybean meal	-	8,0
Soy protein concentrate		14,2
Wheat gluten	-	20,0
Whole wheat	23,8	-
Extruded dehulled peas	-	4,9
Soy-lecithin	-	2,0
DL-Methione	-	0,3
L-Lysine	-	1,4
CaHPO4.2H20	-	3,5
Mineral and vitamin premix**	3,0	3,0
Analysed composition		
Dry matter (DM, % diet)	90,2	92,8
Crude protein (% DM)	46,8	49,4
Crude fat (% DM)	21,6	20,1
Gross energy (kJ g^{-1}DM)	23,7	23,1

*Consisting of (% blend): rapeseed oil (50), palm oil (30), linseed oil (20)
**INRA UPAE, 78352 Jouy-en-Josas, France

Supplementary S2. Mean weights of fish from AMA, APB, RMA, RPB at the start of the experiment. Mean temperature over each 17-day experimental period is reported.

	Start experiment	Day Post-Fertilisation (dpf)	Mean initial weight (g)	Mean temperature (°C)
AMA	29th August	255	58.7 ± 0.7	18.1
APB	10th October	297	82.4 ± 0.6	16.8
RMA	22th August	248	75.9 ± 0.5	17.7
RPB	3rd October	290	83.2 ± 1.3	17.2

Weight values are means and their standard error mean associated (SEM) (n=8)

Supplementary S3. Mean weight (g), survival (%), and weight gain (g), food conversion ratio (FCR) and specific growth rate (SGR) over the rearing period over time for the 4 conditions. FCR and SGR were respectively calculated as:

$$FCR=(F/n)/(w_t-w_{t-1}), \text{ and } SGR=(\log(w_t)-\log(w_{t-1}))/\Delta t*100$$

With F: food provided (g), n the number of fish in the tank, w_t and w_{t-1} respectively the weight (g) at time t and t-1, and Δt the time period.

dpf	condition	Weight (g)	Survival (%)	weight gain (g)	SGR	FCR
72	RMA	0.28	100	0.23	8.61	0.73
72	AMA	0.27	100	0.22	8.43	0.79
72	RPB	0.22	100	0.17	7.33	1.15
72	APB	0.13	100	0.08	4.90	2.21
89	RMA	0.70	98	0.42	5.39	0.54
89	AMA	0.66	97	0.39	5.23	0.88
89	RPB	0.45	96	0.24	4.34	1.12
89	APB	0.23	97	0.10	3.21	2.10
124	RMA	2.65	96	1.95	3.81	0.98
124	AMA	2.29	97	1.63	3.56	1.12
124	RPB	1.51	92	1.06	3.44	1.37
124	APB	1.00	39	0.77	4.21	2.25
151	RMA	6.54	96	3.89	3.34	0.83
151	AMA	4.95	97	2.66	2.86	1.11
151	RPB	3.47	92	1.96	3.08	1.20
151	APB	2.72	39	1.72	3.70	1.02
170	RMA	12.03	96	5.49	3.21	0.68
170	AMA	7.85	97	2.90	2.43	1.11
170	RPB	5.48	92	2.01	2.40	1.37
170	APB	4.53	39	1.80	2.67	1.25
192	RMA	23.70	96	11.68	3.08	0.70
192	AMA	15.55	97	7.70	3.11	0.78
192	RPB	10.68	92	5.20	3.04	0.90
192	APB	8.85	39	4.33	3.05	0.95
214	RMA	46.43	96	22.73	3.06	0.69
214	AMA	29.50	97	13.95	2.91	0.83
214	RPB	21.28	92	10.60	3.13	0.86
214	APB	17.45	39	8.60	3.09	0.94
234	RMA	73.43	95	27.00	2.29	0.82
234	AMA	51.73	97	22.23	2.81	0.79
234	RPB	35.58	92	14.30	2.57	0.96
234	APB	31.05	38	13.60	2.88	0.87

Supplementary S4. Food Conversion Ratio (FCR) and Specific Growth Rate (SGR) over time in two isogenic lines (A and R) of rainbow trout fed a marine (MA) or a plant-based diet (PB). *The mean and error bars are presented for each condition (n=3-4). Lack of vertical bar indicates SE smaller than symbol size.*

FCR and SGR were respectively calculated as:

$$FCR=(F/n)/(w_t-w_{t-1}), \text{ and } SGR=(\log(w_t)-\log(w_{t-1}))/\Delta t*100$$

With F the food provided (g), n the number of fish in the tank, w_t and w_{t-1} respectively the weights (g) at time t and t-1, and Δt the time period.

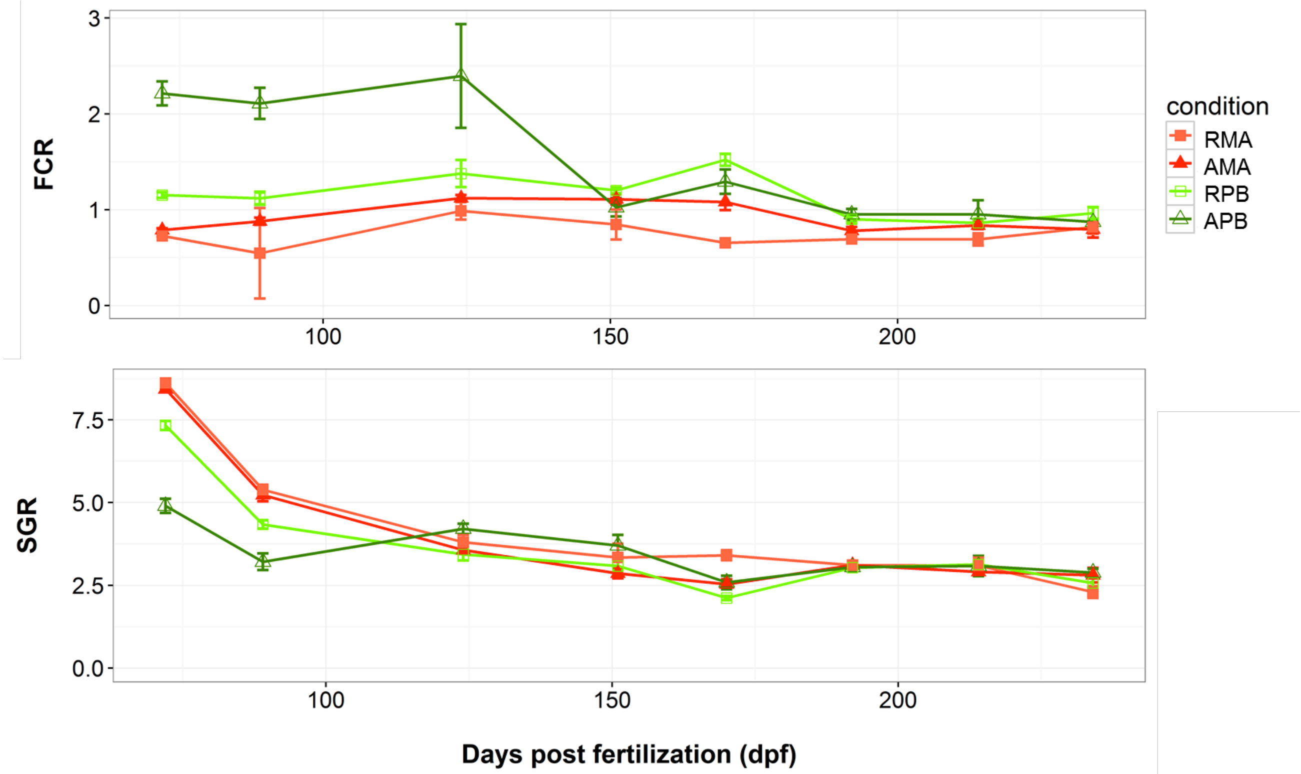

Supplement: Supplementary Information [file srep35957-s1.pdf]
